# 放射性粒子植入治疗肺大细胞神经内分泌癌1例

**DOI:** 10.3779/j.issn.1009-3419.2010.09.16

**Published:** 2010-09-20

**Authors:** 金爽 吕, 树德 柴, 广钧 郑, 卫亮 阎, 震 冯

**Affiliations:** 300211 天津，天津医科大学附属第二医院胸外科 Department of Thoracic Surgery, the 2nd Hospital of Tianjin Medical University, Tianjin 300211, China

大细胞肺癌为肺癌四种主要组织类型中发病率最低的一种^[1]^，其亚型-大细胞神经内分泌癌(large cell neuroendocrine lung cancer, LCNEC)罕见，恶性度高，50%的患者在1年-2年内死亡，5年和10年生存率分别为23%和10%^[2]^。单纯手术治疗效果欠佳^[3]^，目前多采取手术联合化疗为主的综合治疗。天津医科大学附属第二医院应用放射性粒子植入联合化疗诊治患者1例，现报告如下：

## 1 病例资料

患者，男，74岁，因持续左前胸痛、夜间盗汗1个月就诊，既往40余年吸烟史，营养状态欠佳，全身浅表淋巴结无肿大。颈软，气管居中，桶状胸，胸壁无压痛。两侧呼吸活动度对称，叩过清音，双肺可闻及哮鸣音。胸部CT示：左肺上叶前段占位性病变，约2 cm×2 cm(图 1)，透过度增强。肺癌标记物四项：癌胚抗原(carcinoembryonic antigen, CEA) 50.2 ng/mL，肿瘤相关抗原15-3(tumor associated antigen, CA15-3) 23.9 U/mL，铁蛋白432.32 ng/mL，β2微球蛋白3.27 μg/mL；肾素三项：血浆肾素活性0.07 ng/mL/h，血管紧张素54.88 pg/mL，醛固酮6.22 ng/dL；垂体六项：卵泡刺激素(follicle-stimulating hormone, FSH) 11.84 IU/L，促黄体生成激素(luteinizing hormone, LH) 5.9 mIU/mL，促甲状腺激素(thyroid stimulating hormone, TSH) 0.17 μIU/mL，催乳素(prolactin, PRL) 44.27 ng/mL，生长激素(growth hormone, GH) 2.50 ng/mL，促肾上腺皮质激素(adrenocorticotropic hormone, ACTH) 149 pg/mL；降钙素77.57 pg/mL；血浆总皮质醇(plasma total cortisol, PTC) 45.5 μg/dL；骨ECT(-)；肿物活检病理诊断为LCNEC；免疫组化染色：Syn(+)，CgA(+)(图 2)。肺功能测试结果为“中度阻塞性通气功能障碍”。

**1:**
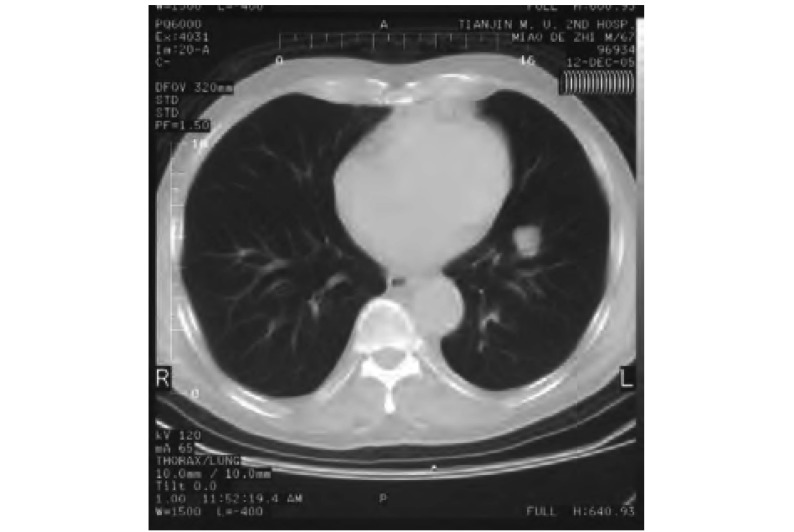
术前胸部CT，LCNEC病灶位于左肺上叶前段 The chest CT scan of the LCNEC preoperative, lesion locate at anterior segmental of the left upper lobe

**2:**
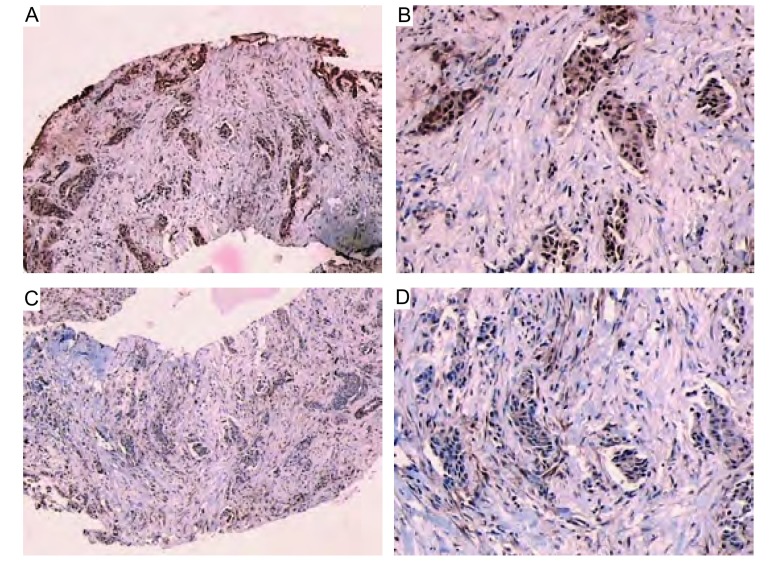
LCNEC患者石蜡切片免疫组织化学染色。A：SYN(×40)；B：SYN(×100)；C：CgA(×40)；D：CgA(×100)。 Immunohistochemical staining of paraffin section in LCNEC. A: SYN (× 40); B: SYN (×100); C: CgA (×40); D: CgA (×100).

鉴于患者年龄大、营养体能状态欠佳和伴有慢性阻塞性肺气肿，决定放弃手术切除而行CT引导下经皮穿刺非血管介入^125^I粒子植入术，联合顺铂+Vp-16化疗及相关辅助治疗。^125^I粒子处方剂量110 Gy，粒子活度0.7 mCi。粒子植入术进行顺利，历时80 min，植入粒子12粒，与术前计划设计的粒子植入数量相同。瘤体接收的平均剂量为226.6 Gy，D_90_ 152.9 Gy，D_100_ 101.5 Gy(图 3)。术中及术后无气胸及肺出血等并发症。植入术后3天给予顺铂+VP-16化疗，每月1次，共6次。术后6个月复查，一般情况好转，胸痛及盗汗减轻，CT见肿物较植入术前缩小60%以上(图 4)。

**3:**
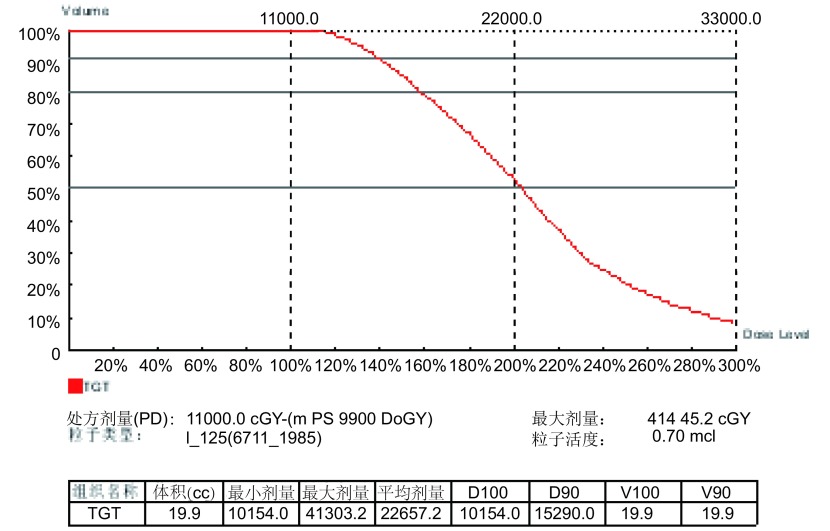
术后处方剂量与肿瘤体积直方图(DVH图)。该图显示瘤体接受的平均照射剂量为226.6 Gy，D_90_ 152.9 Gy，D_100_ 101.5 Gy。 Dose volume histogram (DVH) postoperative. This DVH shows the average radiation dose which tumor received is 226.6 Gy, D_90_ 152.9 Gy, D_100_ 101.5 Gy.

**4:**
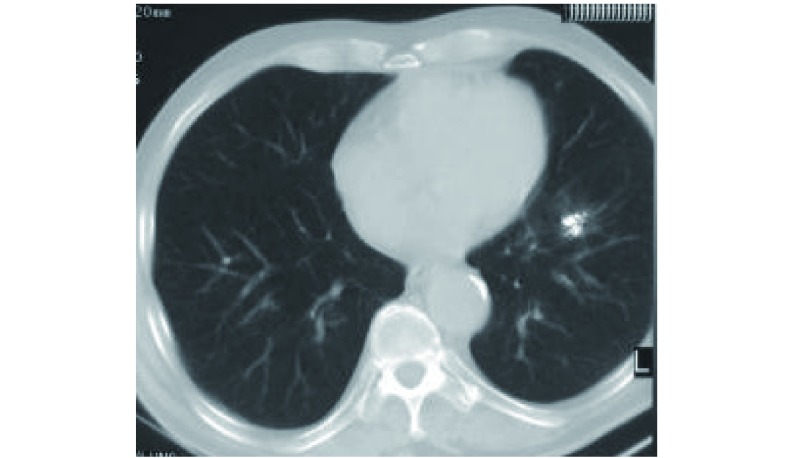
术后6个月胸部CT。胸部CT可见肿瘤较术前缩小60%以上，肿瘤周围出现轻度放射性肺炎改变。 The chest CT scan of 6 months postoperative. This chest CT scan shows the tumor reduced more than 60%, and there is slight change of radiational pneumonitis around it.

## 2 讨论

LCNEC起源于支气管粘膜上普遍分布的Kulchitzky细胞，有神经内分泌肿瘤常见的形态学特征，是一类核状染色质与普通非小细胞肺癌生物学行为有显著区别的病理类型。患者中男性多见，中位年龄62(33-87)岁，大多数均有超过40支/年的吸咽史^[4]^，多数患者表现为无症状的肺部结节、胸痛、非特异性类流感症状、呼吸困难、盗汗以及类癌综合征^[5]^，而副癌综合征并不经常发生^[6-8]^。CT显示周围型占2/3，中央型占1/3，直径0.7 cm-9 cm，平均为4 cm^[1]^。肿瘤通常边界清楚，可见到小分叶，有时可伴边缘毛刺征，结节和肿块内部可见支气管腔、空腔、透亮的空泡或坏死。大约9%的病变内部可见钙化；其它尚包括胸膜牵拽或肿瘤周围肺气肿^[9, 10]^，纵隔淋巴结转移率较高。病理检查通过细胞大小、有无细胞坏死以及有丝分裂频率三个方面与其它类型鉴别^[11]^。神经内分泌肿瘤经常表达生长抑素受体^[12-14]^，故生长抑素受体显像技术对LCNEC有确诊作用^[15]^，相关分子生物学指标的检测也对确立诊断有所帮助。本例患者性别、年龄、吸烟史、胸痛、盗汗、胸CT及肺癌标志物、病理活检等符合本病的诊断。

本例患者应采取肺叶切除，并系统性清扫纵隔淋巴结，辅助化疗或术前放疗等综合治疗^[16]^，但由于患者年龄大、营养情况以及肺功能差，难以耐受根治手术治疗，故对其实施CT引导下经皮穿刺放射性^125^I粒子植入术，辅助顺铂+VP-16化疗。

放射性^125^I粒子植入治疗实体肿瘤的原理是将粒子种植在肿瘤组织内，其持续释放低能γ射线，不断地消耗肿瘤干细胞而使肿瘤细胞死亡。Imamura等^[17]^于1999年首先证实，经皮穿刺插植高剂量率放射性核素治疗肺癌安全有效，无严重副作用。还具有肿瘤靶区高剂量、周围正常组织受量低的特点，弥补了传统外放疗分次短时照射、肿瘤靶区剂量提升困难和对正常组织损伤大的不足^[18]^，本例放射处方剂量110 Gy，瘤体接受的平均照射剂量为226.6 Gy，为处方剂量的2倍，D_90_ 152.9 Gy，D_100_ 101.5 Gy，完全达到肿瘤原位灭活的剂量，是任何外放射治疗剂量难以到达的。术后6个月复查临床症状好转，胸CT显示肿瘤缩小60%以上，目前仍在随访过程中。

CT引导下经皮穿刺种植放射性^125^I粒子治疗肺癌还具有的优点则是局部麻醉微创穿刺，操作时间短，患者痛苦小，恢复快，序贯治疗间隔时间短。操作在CT的监视下进行，保证了粒子植入精确，放射剂量空间分布均匀。对术中发生气胸、肺出血等常见穿刺并发症及时处理，安全可靠。

LCNEC临床罕见，病理学界定复杂，生物学行为特殊，影像学表现多变。对于不能耐受手术及外放疗的患者，采取微创的CT引导下经皮穿刺种植放射性^125^I粒子治疗联合化疗的方法近期疗效明显，不失为一种有效的治疗方式。
